# Expanding the Genetic Code of *Lactococcus lactis* and *Escherichia coli* to Incorporate Non-canonical Amino Acids for Production of Modified Lantibiotics

**DOI:** 10.3389/fmicb.2018.00657

**Published:** 2018-04-06

**Authors:** Maike Bartholomae, Tobias Baumann, Jessica H. Nickling, David Peterhoff, Ralf Wagner, Nediljko Budisa, Oscar P. Kuipers

**Affiliations:** ^1^Department of Molecular Genetics, Groningen Biomolecular Sciences and Biotechnology Institute, University of Groningen, Groningen, Netherlands; ^2^Biocatalysis Group, Department of Chemistry, Technische Universität Berlin (Berlin Institute of Technology), Berlin, Germany; ^3^Institute of Medical Microbiology and Hygiene, Universität Regensburg, Regensburg, Germany

**Keywords:** stop codon suppression, pyrrolysyl-tRNA synthetase, nisin, orthogonal translation system, antimicrobial peptides, bacteriocin, non-canonical amino acids

## Abstract

The incorporation of non-canonical amino acids (ncAAs) into ribosomally synthesized and post-translationally modified peptides, e.g., nisin from the Gram-positive bacterium *Lactococcus lactis*, bears great potential to expand the chemical space of various antimicrobials. The ncAA *N*_ε_-Boc-L-lysine (BocK) was chosen for incorporation into nisin using the archaeal pyrrolysyl-tRNA synthetase–tRNA^Pyl^ pair to establish orthogonal translation in *L. lactis* for read-through of in-frame amber stop codons. In parallel, recombinant nisin production and orthogonal translation were combined in *Escherichia coli* cells. Both organisms synthesized bioactive nisin(BocK) variants. Screening of a nisin amber codon library revealed suitable sites for ncAA incorporation and two variants displayed high antimicrobial activity. Orthogonal translation in *E. coli* and *L. lactis* presents a promising tool to create new-to-nature nisin derivatives.

## Introduction

**FIGURE 1:**
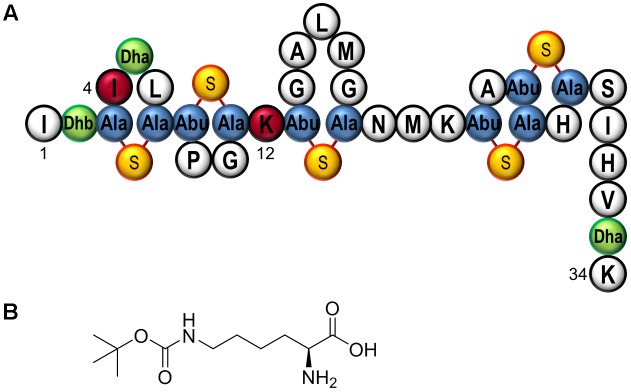
**(A)** Schematic structure of fully modified WT nisin. This form of the antimicrobial peptide lacks the N-terminal leader, which has been proteolytically cleaved by NisP, activating the lantibiotic as the last maturation step. The 34 amino acid peptide is displayed in the conventional numbering scheme from core peptide position I1 until K34. Black circles represent canonical amino acids in the one letter code and green filled circles represent dehydroalanine (Dha) or dehydrobutyrine (Dhb). Blue filled circles depict the (methyl)lanthionine rings comprising alanine (Ala) or aminobutyric acid (Abu) with thioether bridges depicted by yellow–orange circles. The analyzed locations for ncAA incorporation, nisinI4 and nisinK12, are highlighted in red. **(B)** Structure of *N*_ε_-Boc-L-lysine (BocK), the ncAA utilized in this study.

Lantibiotics represent a class of peptide antibiotics that shows promising antimicrobial activity against Gram-positive pathogens, e.g., *Staphylococcus aureus* or *Clostridium difficile* (Castiglione et al., 2007; Bierbaum and Sahl, 2009). The best studied example is nisin, synthesized by the Gram-positive bacterium *Lactococcus lactis*. It belongs to the class of RiPPs and is translated as a precursor comprising a 23 amino acid leader peptide fused to a 34 amino acid core peptide. The leader serves as a docking station for the modification enzymes, directs the transport of the peptide out of the cell, and keeps it inactive to prevent activity against the producer strain (Bierbaum and Sahl, 2009; Ortega et al., 2015). Within the core peptide, specific serine and threonine residues are dehydrated by the dehydratase NisB to form dehydroalanine (Dha) and dehydrobutyrine (Dhb). In a cyclization reaction catalyzed by the cyclase NisC, these unusual amino acid derivatives are combined with the next downstream cysteine to form the five characteristic (methyl)lanthionine rings which are essential for bioactivity. The post-translationally modified peptide is transported out of the cell by the transporter NisT. Finally, the leader peptide is cleaved off by the membrane-associated protease NisP, to release active nisin (see **Figure 1A** for a schematic structure of fully modified active nisin) (Lubelski et al., 2008).

The main characteristics of all RiPPs are the PTMs. These are crucial, since an unmodified prepeptide lacks antimicrobial activity (Arnison et al., 2013). The natural variety of modifications is tremendous, from simple terminal decapping to complex ring formation in nisin (Wang, 2012). Complete chemical synthesis of nisin is feasible, however, since the molecular structure is highly complex, it suffers from a tedious multi-step synthesis and low yield (Fukase et al., 1988; Ongey and Neubauer, 2016). Therefore, *in vivo* methods for engineering RiPPs are required.

One promising approach is the incorporation of ncAAs, since, apart from the 20 standard proteinogenic L-amino acids, they offer new chemical features to AMPs, e.g., by introducing fluorinated or photocaged residues (McKay and Finn, 2014; Zambaldo et al., 2017). Moreover, this approach provides a whole toolkit of new chemical modifications for AMPs, which even allows to combine peptides by diverse “click chemistry” approaches, e.g., the Staudinger ligation (Wang et al., 2015) or strain-promoted azide-alkyne cycloaddition (SPAAC) (Budisa, 2013; Kim et al., 2016).

The pharmaceutical potential of ncAAs – in form of free amino acids, as well as a modification of peptides and proteins – has been reviewed recently (Blaskovich, 2016). Various examples demonstrate the potential of ncAA-modified linear AMPs. For example, the incorporation of proline analogs into proline-rich peptides increased their macrophage penetration potential and their activity against *Listeria*, *Brucella*, or methicillin-resistant *S. aureus*. Moreover, the stability of the corresponding AMPs against proteolytic degradation by trypsin has been improved (Kuriakose et al., 2013). First studies with nisin revealed a change in its antimicrobial activity if it was supplemented with either tryptophan- or proline analogs at different locations by force-feeding (Zhou et al., 2016b; Baumann et al., 2017). A drawback of this method is the global replacement of one canonical amino acid by the chosen ncAA in the whole proteome of the producing cell (Hoesl and Budisa, 2012; Zhou et al., 2016a; Baumann et al., 2017).

To specifically introduce the ncAA at the desired position in the protein of interest, we focused on implementing the SCS method (Wang et al., 2001) in *L. lactis*. This technology allows reprogramming the amber stop codon TAG to a sense codon for the ncAA. Genome analysis revealed TAG to be the least frequently used stop codon in *L. lactis* (Gupta et al., 2004). Consequently, introduction of an OTS which is based on amber suppression was anticipated to have a low impact on host cell fitness. As key components, the pyrrolysine (Pyl) tRNA synthetase and its corresponding tRNA from the archaeon *Methanosarcina mazei* [pyrrolysyl-tRNA synthetase (PylRS)–tRNA^Pyl^] are commonly utilized. This aminoacyl-tRNA synthetase recognizes its cognate tRNA and naturally charges it with the 22nd amino acid Pyl, allowing its ribosomal incorporation into the growing polypeptide chain. This enzyme is of particular interest in biotechnology because of its high substrate promiscuity resulting from a relatively unspecific amino acid recognition by mainly hydrophobic interactions, which allows incorporation of various new amino acid variants (Wan et al., 2014; Tharp et al., 2017). Being orthogonal in bacterial and eukaryotic cells, PylRS is commonly used for incorporation of ncAAs, not only in *Escherichia coli*, but also in yeast (Hancock et al., 2010) and mammalian cells (Mukai et al., 2008). Recently, SCS was established in *Bacillus cereus* as the first Gram-positive organism (Luo et al., 2016) and in *Synechococcus elongatus* as the first photoautotrophic cyanobacterium (Chemla et al., 2017).

In this study, a double tracked approach toward ncAA-modified nisin was followed: First, *E. coli* was equipped with both the SCS and nisin biosynthesis machineries for recombinant production of nisin modified with *N*_ε_-Boc-L-lysine (BocK) as chosen ncAA (**Figure 1B**). Second, the genetic code of *L. lactis*, the Gram-positive native nisin production host, was expanded by introducing the PylRS–tRNA^Pyl^ pair to enable SCS. Following site-specific incorporation of BocK into different locations of nisin *in vivo*, the effects on the antimicrobial activity of nisin were analyzed.

## Materials and Methods

Bacterial strains, plasmids, and oligonucleotides are described in Supplementary Tables S1 and S2. Experiments on ncAA incorporation were independently conducted at least twice in order to ensure the reproducibility of the data.

### Generation of an Amber Codon-Scanned Library of *nisA*

An amber codon-scanned library of *nisA* with all sense codons of the core peptide individually replaced by TAG was generated via multiple parallel overlap extension PCR reactions (Ho et al., 1989). A synthetic construct pET-21a_P_His6_leader_nisA served as a template. This construct contains the WT sequence of *nisA* (GenBank: HM219853.1) together with an N-terminal His-tag fused by a small linker to the coding sequence (complete encoded amino acid sequence: MGSSHHHHHHSQDP). Primers used for the generation of the library are listed in Supplementary Table S2. The individual *nisA*(amber) variants were cloned via NdeI and XhoI restriction sites into the expression vector pET-21a (Merck).

For expression in *E. coli*, selected variants from the *nisA*(amber) library (flanked by a T7 promoter and terminator) were PCR-amplified with the primers PT7-NisA_f and NisA T7term_r. Purified PCR products were cloned using SpeI and PstI into pJZ_Ptrp_pylT_strep-MmPylS(Y384F) treated with XbaI and PstI to yield pJZ_Ptrp_pylT_MmPylS(Y384F) PT7 nisA(amber). In order to combine PTM and SCS for the production of ncAA-modified nisin variants, BL21(DE3) cells were co-transformed with the latter plasmid and pRSFDuet-1 nisBC.

To express tRNA^Pyl^ and PylRS in *L. lactis*, *pylTS* were amplified from plasmid pJZ_Ptrp_pylT_strep-MmPylS(Y384F) with the primers PylTSfwNco and PylTSrevKpn. The PCR product and the vector pLG_ΔRBS_-GFP were digested with the restriction enzymes NcoI and KpnI and ligated, resulting in vector pNZ-RBSpylTS. Subsequently, selected *nisA*(amber) variants were amplified by the primer pair NisLibraryfwKpn and NisLibraryrevXba. The resulting PCR products and the vector pNZ-RBSpylTS were digested by KpnI and XbaI, and the fragments were assembled to form plasmid pNZ-RBSpylTSnisA(amber). To synthesize the *nisA* WT control, the amber codon of pET-21a_P_His6_leader_nisA(K34amber) was replaced by the WT lysine codon using the mutagenesis primer NisAWTrevXba, while as forward primer NisLibraryfwKpn was utilized. The further cloning procedure was identical and resulted in the plasmid pNZ-RBSpylTSnisA. To enable the PTMs of the nisin precursor (dehydration and cyclization followed by transport out of the cell), *L. lactis* NZ9000 pIL3EryBTC encoding the modification enzymes NisBTC was transformed by the *nisA* plasmid constructs.

### Production of ncAA-Containing Nisin Variants in *E. coli*

Precultures of *E. coli* BL21(DE3) pJZ_Ptrp_pylT_MmPylS (Y384F) PT7 nisA(amber) pRSFDuet-1 nisBC were grown at 37°C in LB medium supplemented with antibiotics and 1% w/v glucose. Target gene expression was conducted at 27.5°C overnight in autoinducing ZYP-5052 medium (Studier, 2005) supplemented with antibiotics. Next, bacterial cells were harvested by centrifugation. Purification of nisin variants was conducted as described earlier using an N-terminally His-tagged peptide leader and Ni-NTA affinity columns (Baumann et al., 2017).

### Preparation of Nisin Variants Modified by ncAAs from *L. lactis*

*Lactococcus lactis* NZ9000 pNZ-RBSpylTSnisA(amber) pIL3EryBTC was grown in CDM medium (containing 5 μg/ml each chloramphenicol and erythromycin) to an OD_600_ of 0.4. Overexpression was induced with 10 ng/ml nisin and the medium was supplemented with 1-5 mM *N*_ε_-Boc-L-lysine (BocK) to grow for another 3 h. After harvesting, the supernatant was filtered, acidified, and finally purified by ion exchange chromatography using a 5 ml HiTrap SP-Sepharose (GE Healthcare) column. Fractions showing antimicrobial activity were pooled, desalted, and finally freeze-dried. For HPLC purification on an Agilent 1260 Infinity LC instrument, samples showing antimicrobial activity were desalted with a Sephadex G10 column (GE Healthcare) and applied to a C12 column (Phenomenex 250 × 4.5 mm, 4 μm, Proteo 90Å) as described earlier (Zhou et al., 2016b).

### Agar Well Diffusion Assay

To determine the antimicrobial activity of nisin and ncAA-modified nisin(BocK) variants synthesized by *L. lactis*, solid medium was inoculated with 100 μl of nisin-sensitive *L. lactis* NZ9000 pNZnisPT pIL253 overnight culture. The first plasmid (pNZnisPT) leads to expression of NisP, which can activate the nisin variants later added to the agar by proteolytic cleavage of the leader peptide. Plasmid pIL253 is an “empty” control plasmid conferring erythromycin resistance, thus avoiding growth inhibition from potential carryover of the antibiotic from the *L. lactis* production strain supernatant. Fifty microliters of either filtered culture supernatant or HPLC-purified peptides was applied to the testing wells (diameter: 7.5 mm) and incubated at 30°C overnight, until inhibition halos were visible.

For nisin variants produced by *E. coli*, antimicrobial activity tests were conducted as described (Baumann et al., 2017), using the same nisin-sensitive and NisP-expressing *L. lactis* indicator strain. Briefly, cell lysates produced from 1 ml bacterial culture were normalized by harvested cell density (OD_600_) using PBS buffer. Alternatively, peptide samples purified by immobilized metal ion affinity chromatography (IMAC) were used. Chloramphenicol at 400 μg/ml was used as antibacterial control compound.

### Mass Spectrometry

For MALDI–TOF–MS analysis, 1 μl of HPLC-purified sample was applied to the matrix target and treated as described earlier on a Voyager DE Pro MALDI-TOF spectrometer (Applied Biosystems) (van Heel et al., 2013). Data analysis was carried out with “Data Explorer” software version 4.0.0.0 (Applied Biosystems). Calculation of theoretical masses with ncAA-modified nisin variants was carried out with “massXpert”, version 3.4.0 (Rusconi, 2009).

For LC–ESI–TOF–MS analysis, IMAC-purified samples were analyzed using a QTOF 6530 instrument (Agilent) as described (Baumann et al., 2017).

### Immunoblotting

Pyrrolysyl-tRNA synthetase expression was verified via an added N-terminal Strep-tag. After cell growth and harvesting, cell pellets were dissolved in PBS buffer (58 mM Na_2_HPO_4_, 17 mM NaH_2_PO_4_, 68 mM NaCl) and ruptured by bead-beating as described earlier (Neves et al., 2010). Strep-PylRS was purified with Strep-Tactin^®^ Resin (IBA). Fractions containing PylRS were selected by SDS-Page and verified by immunoblotting with a 1:100 diluted Strep-Tactin HRP (IBA) conjugate according to manufacturer information. HRP activity was detected using the Amersham ECL Prime Western Blotting Kit (GE Healthcare).

## Results

### Combining Stop Codon Suppression (SCS) and Production of Post- translationally Modified Nisin in *E. coli*

In a previous study, recombinant production of fully modified nisin was established in *E. coli* (Shi et al., 2011). Based on this data, we constructed a T7 promoter-based setup for the recombinant expression of *nisABC*. The expression of *nisA*, *nisBC*, and *pylTS* from three different plasmids revealed to be a significant metabolic burden for the BL21(DE3) host cells, resulting in slow growth and a low final optical density of the cultures (data not shown). Thus, we reduced the number of required plasmids by introducing *nisA*(amber) variants into the OTS plasmid yielding pJZ_Ptrp_pylT_MmPylS(Y384F) PT7 nisA(amber). We decided to explore a full amber scanning library, which displays in-frame amber stop codons successively replacing each of the sense codons of the nisin core peptide, to allow for identification of variants with efficient SCS and retaining activity. Following plasmid construction, *E. coli* was co-transformed by the individual library member plasmids and a compatible second plasmid for NisBC co-expression. The latter two PTM enzymes catalyze the dehydration and circularization of the nisin precursor peptide (Lubelski et al., 2008). Using autoinduction medium, *nisABC* expression was driven by the T7 promoter system, whereas OTS expression (*pylTS*) was constitutive.

**FIGURE 2:**
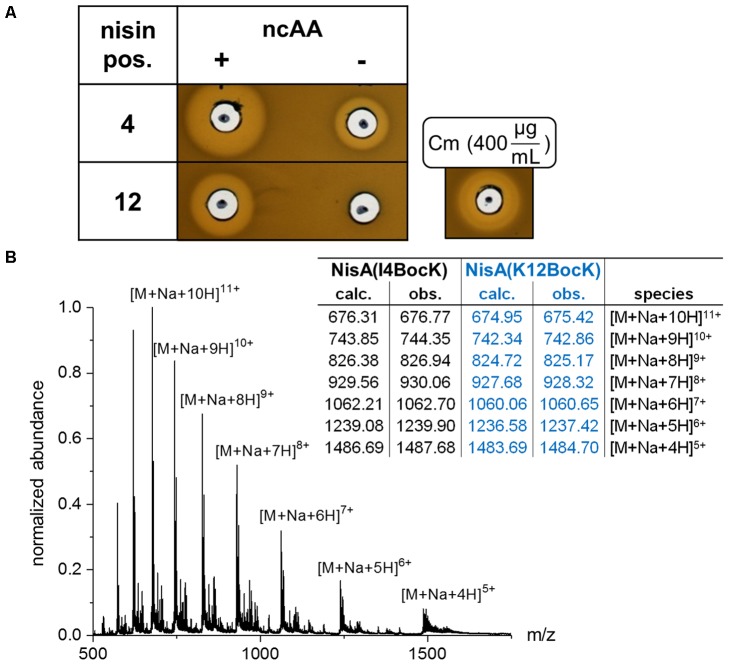
Antimicrobial activity assay and LC–MS analysis of ncAA-modified nisin produced by SCS in *E. coli.* **(A)** Antimicrobial activity of recombinantly produced nisin variants against the nisin-sensitive indicator strain *L. lactis* NZ9000 pNZnisPT pIL253 (this strain expresses NisP, which catalyzes removal of the nisin leader peptide). As indicated, recombinant expression of both *nisA*(amber) variants was conducted in presence or in absence of BocK. Fifty microliters of *E. coli* cell lysate normalized by harvested cell density was used. Cm served as antimicrobial control compound. **(B)** ESI–MS deconvolution chromatogram for nisin(I4BocK) and nisin(K12BocK) samples purified via IMAC. Theoretical [M+Na]^+^ masses for the ncAA-modified prepeptides (still carrying the leader) after PTM by NisBC are 7428.46 and 7413.45 Da, respectively. Observed masses: 7428.40 and 7413.47 Da. Chromatogram shown for nisin(I4BocK), see Supplementary Figure S3B for that of nisin(K12BocK).

Following recombinant expression of *pylTS* and *nisABC* in *E. coli*, conducted both in presence and in absence of BocK (**Figure 1B**), the cell lysates were examined for antimicrobial activity. For this, the nisin-sensitive NisPT-expressing *L. lactis* strain NZ9000 pNZnisPT pIL253 (Khusainov et al., 2011) was used, which is an indicator strain capable to cleave the leader peptide to yield fully mature active nisin. Although mature WT nisin is ineffective against the Gram-negative bacterium *E. coli* (Helander and Mattila-Sandholm, 2000), a *nisA* leader carrying an N-terminal His-tag was utilized in order to facilitate purification of the produced peptides. This modification does not influence the removal of the leader peptide, since the WT NisP cleavage sequence ASPR | IT remains intact (Plat et al., 2011; Lagedroste et al., 2017; Montalban-Lopez et al., 2018). Screening of the amber-scanned *nisA* library was performed. Targeting residues corresponding to the first two rings in the mature lantibiotic (i.e., I1 until K12, compare **Figure 1A**) revealed promising candidates, where ncAA-dependent antimicrobial activity was observed. Distinct inhibition of microbial growth was observed for samples corresponding to nisin(I4BocK) and nisin(K12BocK), whereas very low or no inhibition, respectively, took place in absence of ncAA supplementation (**Figure 2A**). Noteworthy, SCS at most positions within the prepeptide did not result in bioactivity (Supplementary Figure S2). With no or little change upon ncAA (BocK) addition, activity was also observed when the amber stop codon was placed close to the 3′-end of *nisA*, as shown for the penultimate prepeptide position in construct *nisA*(S33amber) (Supplementary Figure S2). The latter is in line with observations made from production of modified/truncated variants in *L. lactis*: also those truncated prepeptides become processed by NisBC *in vivo* and attain detectable levels of antimicrobial activity (Rink et al., 2007).

Next, ncAA-modified peptides were purified via IMAC, utilizing the N-terminally His-tagged nisin leader. For both the WT nisin as control and the SCS samples, antimicrobial activity was detected from elution fractions and concentration of bioactive nisin was evident as judged from the size of inhibition halos (Supplementary Figure S3A). Consequently, IMAC purification yielded samples of improved purity and antimicrobial activity. LC–MS analysis was conducted to verify incorporation of the ncAA into the RiPP. Likewise, the WT construct without an in-frame amber stop codon was recombinantly produced, purified, and analyzed. As anticipated, purified peptide fractions were found to contain NisBC-processed [i.e., (Me)Lan-containing, cyclized] nisin, for the SCS samples carrying BocK at positions 4 and 12, respectively (see **Figure 2B** and Supplementary Figure S3B for theoretical and observed molecular masses).

Following established procedures, efforts were made to quantify and optimize ncAA-containing nisin variant production. Via Coomassie-stained PAGE, it was observed that peptide quantities and purities were lower than reported earlier (Shi et al., 2011) (data not shown). Yields of recombinant production were reduced upon ncAA incorporation as commonly is the case for SCS (Zheng et al., 2016). It should be noted that recombinant production and SCS were performed in a release factor-1 (RF-1) positive *E. coli* B laboratory strain [BL21(DE3)], where amber SCS competes with translation termination. Nevertheless, the PylRS-based system can deliver efficient amber suppression in this strain as long as a single in-frame stop codon is used (Odoi et al., 2013). Furthermore, *E. coli* BL21(DE3) was chosen as it grows robustly to high cell density in the nisin biosynthesis setup (data not shown). Despite the genetic complexity of the system, the feasibility of recombinant ncAA-containing nisin production was demonstrated. Employing the WT *L. lactis* PTM enzymes for prepeptide processing, positions were identified to allow for BocK incorporation into nisin – leading to novel bioactive ncAA-modified RiPPs. Since nisin(I4BocK) and nisin(K12BocK) represented reasonable antimicrobial activity, they were chosen as candidates to transfer this OTS into the natural production host of nisin, *L. lactis*.

### Modification of P_nisA_ Enables SCS by PylRS–tRNA^Pyl^ Expression in *L. lactis*

**FIGURE 3:**
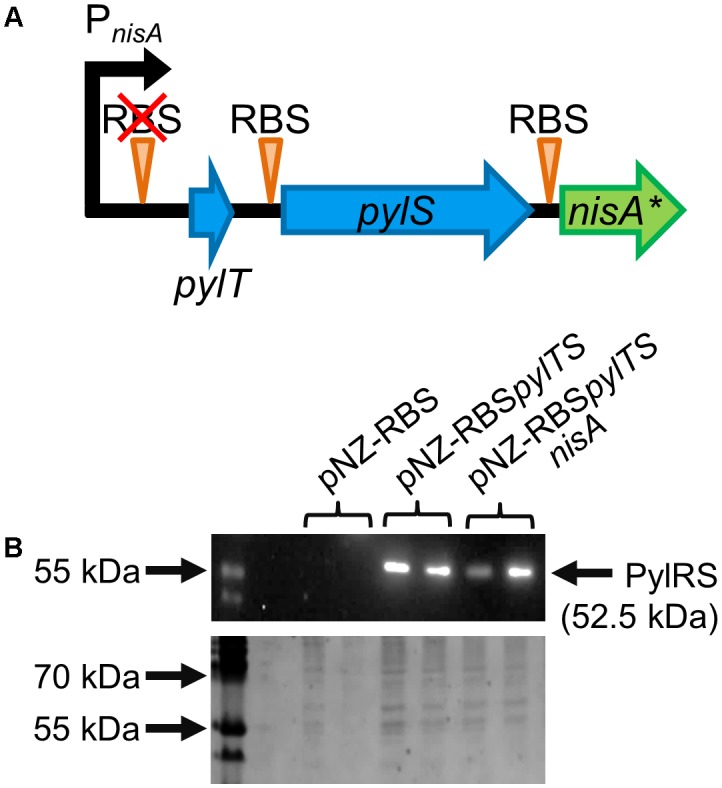
Modification of the nisin promoter P_nisA_ for combined OTS and nisin expression in *L. lactis.* **(A)** Modified P_nisA_ (black arrow) of vector pNZ-RBSpylTS and its derivatives with deleted RBS upstream of *pylT* (encoding tRNA^Pyl^) to prevent initiation of translation. RBS locations are indicated by orange triangles, the original RBS location is crossed out. In blue arrows, the components of the OTS, *pylT* and *pylS* are depicted. The green arrow labeled with *nisA^∗^* represents the nisin precursor gene, either encoding the WT (*nisA*) or variants with in-frame stop codons [*nisA*(amber)]. The size of the depicted genes is not drawn to scale. **(B)** Heterologous expression of archaeal PylRS controlled by modified P_nisA_. Cell extracts from *L. lactis* pNZ-RBS, pNZ-RBSpylTS, and pNZ-RBSpylTSnisA were applied to a Streptactin column and the two elution fractions were analyzed by immunoblotting with a Streptactin–HRP conjugate. Only cells expressing PylRS led to detection by the antibody (upper picture). Strep-tagged Page Ruler Standard (Thermo Fisher Scientific) served as positive control, apparent molecular weight and calculated value for PylRS are indicated. The corresponding 10% SDS-PAGE stained with Coomassie brilliant blue revealed that all analyzed fractions contained protein (picture below).

Establishing a functional PylRS-based OTS in *L. lactis* by utilization of the NICE system (Kuipers et al., 1998) required a modification of the nisin inducible promoter P_nisA_. The ribosomal-binding site (RBS) located within the WT P_nisA_ was disrupted by mutating the sequence AAGGAG to AATTCG (van Gijtenbeek et al., 2016) to hinder unwanted translation of *pylT* encoding the orthogonal tRNA. As depicted in **Figure 3A**, an additional RBS was added upstream of *pylS* as well as of *nisA*(amber). Despite these genetic rearrangements, the new variant of P_nisA_ remained functional, because expression of N-terminally Strep-tagged PylRS was detectable by Streptactin immunoblotting. After induction of PylRS expression by nisin, cell extracts of *L. lactis* NZ9000 pNZ-RBSpylTS and pNZ-RBSpylTSnisA as well as the empty vector pNZ-RBS as negative control were applied to a Streptactin column. After purification, immunoblotting revealed a band of matching molecular weight by a Streptactin HRP conjugate in elution fractions resulting from PylRS expressing cells in contrast to cells bearing the control vector (**Figure 3B**).

### Nisin Bearing ncAAs

With inducible PylRS expression confirmed, the functionality of PylRS–tRNA^Pyl^ in *L. lactis* was determined with a GFP amber suppression reporter. The detailed experimental setup is available in the Supplementary Material, Section 1. In short, the fluorescence of the GFP variants produced in the presence and absence of BocK was determined by FACS and compared to cells expressing WT GFP as positive control and cells expressing only PylRS–tRNA^Pyl^ as negative control. The experiments revealed a small, but reproducible increase of the intact cell fluorescence in the presence of BocK, documenting the functionality of the OTS resulting in incorporation of BocK into GFP (Supplementary Figure S1).

**FIGURE 4:**
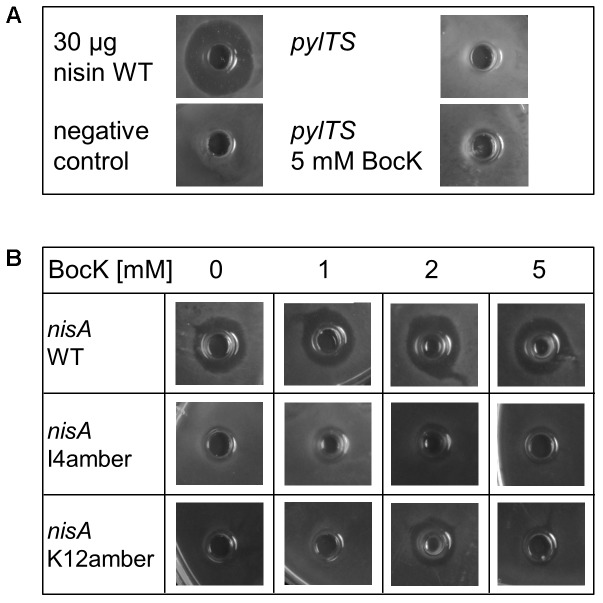
Antimicrobial activity of nisin variants produced in *L. lactis*. Samples were tested for growth inhibition of the nisin-sensitive indicator strain *L. lactis* NZ9000 pNZnisPT pIL253. The producer strains lack NisP, so that produced nisin variants remain inactive since the leader peptide stays attached. Upon addition of cell lysate or purified peptide samples to the NisP-expressing indicator strain, active nisin can be liberated. **(A)** Control samples: 30 μg pure nisin was used as positive control and pure CDM medium as negative control. To exclude antimicrobial activity resulting from PylRS–tRNA^Pyl^ expression or addition of 5 mM BocK to the medium, the corresponding supernatant samples were tested separately and showed no antimicrobial activity. **(B)** Comparison of antimicrobial activity of WT nisin and nisin(BocK) variants produced in presence of different BocK concentrations. The rising BocK concentrations did not affect the antimicrobial activity of nisin WT. In the absence of BocK, expression of *nisA*(I4amber) or *nisA*(K12amber) did not result in antimicrobial activity. Only upon addition of up to 5 mM BocK, antimicrobial activity was observed, with 2 mM as optimal concentration.

To determine if the same ncAA can also be incorporated into nisin, *gfp*(amber) was replaced by *nisA*(amber). As target locations in nisin, the codons for core peptide I4 and K12 were chosen, as they were promising candidates as indicated by the *E. coli* experiments (see above). To synthesize the variants and to purify them from the culture supernatant, *L. lactis* NZ9000 transformed with pIL3EryBTC and pNZ-RBSpylTSnisA(I4amber) or pNZ-RBSpylTSnisA(K12amber), respectively, was cultivated. These strains express the transporter NisT capable to transport nisin out of the cells, which enables peptide purification from the culture supernatant. The nisin variants remain inactive at this point, because no protease is present to cleave off the leader peptide. The antimicrobial activity of the supernatant of the corresponding cultures was checked against the nisin-sensitive indicator strain *L. lactis* NZ9000 pNZnisPT pIL253. By using this NisP producing indicator strain, the leader peptide gets cleaved off, liberating active nisin (Khusainov et al., 2011). As seen before for samples purified from *E. coli*, only samples from production cultures supplemented with BocK showed antimicrobial activity, indicating that translation was terminated in absence of ncAA supplementation and that no canonical amino acid was incorporated instead. Three internal controls were utilized: cells bearing pNZ-RBSpylTSnisA pIL3EryBTC served as positive control to determine the influence of the rearranged promoter on nisin WT production. Culture supernatant of these cells always showed antimicrobial activity independent of BocK addition, assuring the general functionality of the nisin production system. Two internal negative controls were performed. Supernatant of cells expressing only PylRS–tRNA^Pyl^ either in the presence and absence of BocK never had any antimicrobial effect. Consequently, antimicrobial activity was caused by the novel nisin variants and not by the orthogonal tRNA synthetase–tRNA pair or the supplemented ncAA (**Figure 4**).

**FIGURE 5:**
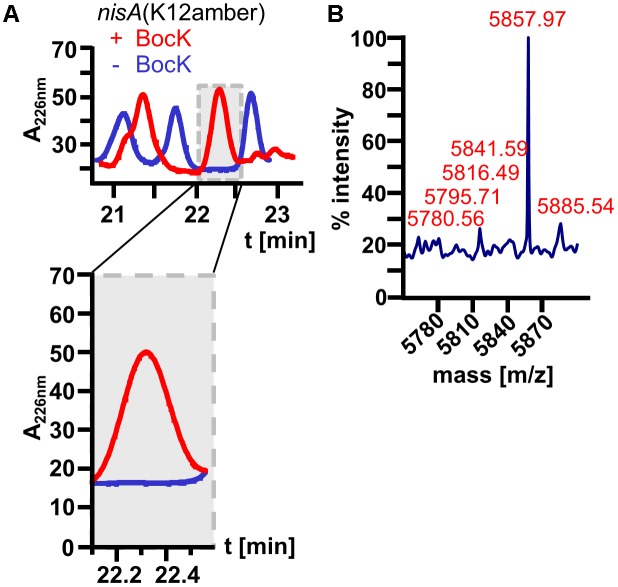
HPLC purification of nisin(K12BocK) (red) produced in *L. lactis* and the corresponding control produced in the absence of BocK supplementation nisin(K12amber) (blue). **(A)** Comparing the elution chromatograms, the most interesting peak at 22 min is zoomed in (gray shading). Only if BocK was available in the growth medium, the highlighted peak was detectable. In contrast, no peak was visible for the control which lacks BocK supplementation (blue), indicating that no full-size peptide was synthesized. **(B)** MALDI–TOF–MS analysis of the samples above picture exemplary for all other measurements described in **Table 1**. For nisin(K12BocK), several different dehydration variants were detected. The most prominent signal at *m*/*z* = 5857.97 indicates a nisin variant with only four dehydrations. Depending on the location of the dehydrated serines and threonines, the formation of the first two or three rings is still possible, resulting in antimicrobial activity of the variants.

For further proof of ncAA incorporation into nisin, HPLC-purified nisin(BocK) samples were analyzed by MALDI-TOF mass spectrometry. These measurements revealed that ncAA-modified nisin variants were synthesized at a very low level with the correct molecular weight (**Table 1**). Surprisingly, always a mixed population of full-size nisin(BocK) samples was determined: a larger one without His-tag and a smaller one with His-tag was found (data not shown). In the chosen genetic setup, the *nisA* reading frame starts with the ATG start codon of the N-terminal His-tag and contains a second, internal ATG codon originating from the first methionine of the leader, so two peptide variants (resulting from two different translation initiation sites) are possible. Selection of the untagged variant by the chosen purification method, differing stabilities of His-tagged and untagged variant or different folding of the peptide variants during biosynthesis in *L. lactis* are possible explanations for this phenomenon. Additionally, for nisin(I4BocK) and nisin(K12BocK), peaks corresponding to different dehydration states of the peptide were identified. Still, the main detected dehydration state corresponds to a nisin molecule with two or three rings, which explains the observed antimicrobial activity (Rink et al., 2007). As control, HPLC-purified peptide samples from cells grown without ncAA supplementation were utilized. As expected, MALDI-TOF analysis showed no peaks corresponding to full-size peptide products for this control (**Figure 5**).

**Table 1:** Determination of the molecular weight of WT and nisin(BocK) variants produced in *L. lactis* by MALDI–TOF–MS.

Nisin variant	Modification^∗^	Predicted mass (Da)	Observed mass (Da)
Nisin WT	–8 H_2_O, –M	5585.69	5588.92
Nisin(I4BocK)	–8 H_2_O, –M	5718.75	5714.67
	–7 H_2_O, –M	5736.75	5735.2
Nisin(K12BocK)	–8 H_2_O, –M	5784.81	5780.56
	–7 H_2_O, –M	5802.81	5795.71
	–6 H_2_O, –M	5820.81	5816.49
	–5 H_2_O, –M	5838.81	5841.59
	–4 H_2_O, –M	5856.81	5857.97
	–2 H_2_O, –M	5892.81	5885.54


*^∗^–M, removal of the N-terminal initiator methionine residue.*

### Expression of *nisA*(amber) in the Presence of Different Concentrations of BocK

To elaborate if increased ncAA concentrations can improve the production of nisin(BocK) variants, *L. lactis* cultures were supplemented with 0, 1, 2, and 5 mM BocK. As expected, the antimicrobial activity of WT nisin was not influenced by different BocK concentrations. Interestingly, for all tested nisin(BocK) variants, 2 mM BocK delivered the highest antimicrobial activity. Again, MALDI-TOF analysis confirmed the correct peptide mass (compare **Figure 5**). In conclusion, incorporation of ncAAs into nisin with *L. lactis* as expression host is possible and highly specific. Still, the *in vivo* synthesized amount of the ncAA-modified RiPP variants needs further improvement. Despite the low peptide yields, we could demonstrate the functional transfer of the PylRS-based SCS machinery to the Gram-positive host *L. lactis.*

## Discussion

In this study, we established genetic code expansion of *L. lactis* by functional expression of an OTS based on PylRS–tRNA^Pyl^. The successful combination of SCS and PTM enzymes led to the production of bioactive BocK-modified nisin variants. To identify the ideal host for genetic code expansion and post-translationally modified lantibiotic production, we tested two expression hosts: *E. coli* and *L. lactis*, because each of them has distinct advantages.

Since SCS represents a method predominantly described for *E. coli* strains with single (Wang et al., 2001) or multiple ncAA incorporation and with PylRS expression adopted to this expression host (Sugawara and Nikaido, 2014; Baumann et al., 2018), it was at first glance the promising host of choice for realizing ncAA-modified nisin. Additionally, in-depth analysis of PylRS–tRNA^Pyl^ expression in *E. coli* revealed CUA as the ideal tRNA^Pyl^ anticodon for ribosomal incorporation of BocK into growing polypeptide chains (Odoi et al., 2013). The production of post-translationally modified active nisin in *E. coli* was already documented several times (Shi et al., 2011; Baumann et al., 2017). Moreover, the type II lantibiotic haloduracin was expressed with the required PTM enzymes and in parallel, *p*-benzoyl-L-phenylalanine was incorporated by SCS (Shi et al., 2011). Recently, the first SCS approach with nisin in *E. coli* was documented (Zambaldo et al., 2017). In contrast to our general approach which aimed to find the optimal ncAA location resulting in the highest antimicrobial activity, the latter focused only on certain selected serine positions. Aiming for the construction of new ring topologies, these serines were replaced by phenylalanine analogs. However, this did not yield bioactive molecules. In our case, the obtained new-to-nature nisin(BocK) variants were bioactive and modified by both the ncAA and the natural PTM machinery.

Due to the complexity of the methodology, also the challenges of this method were analyzed in depth in *E. coli*. Previous studies gave insights into possible reasons for low production yields using an OTS: The kinetics of amino acid activation and tRNA charging by PylRS gave information about the efficacy of the enzyme. Relative to canonical aminoacyl tRNA synthetases, its *k*_cat_ values are about 1000 times lower. In the natural context, this turnover rate of the enzyme is sufficient, since in *Methanosarcina* only around 50 Pyl (amber) codons are found in all transcribed genes. In contrast, in *E. coli* the leucyl-tRNA synthetase needs to provide substrates for approximately 150,000 codons. Relative to the already low efficiencies for the WT substrate Pyl, variants engineered for ncAA incorporation commonly perform worse (Guo et al., 2014). Certainly, SCS brings along a reduction in RiPP production yields, since five recombinant genes of different origins have to be expressed: *pylTS* from archaeal alongside *nisABC* from Gram-positive origin, all in a Gram-negative host organism. Combined with the metabolic burden to maintain the corresponding plasmids, this illustrates the challenge of combining SCS with RiPP synthesis (Ow et al., 2006).

Since *nisABTC* originate from *L. lactis* and most lantibiotics are originally synthetized by Gram-positive bacteria (Dischinger et al., 2014), *L. lactis* as expression host for PylRS–tRNA^Pyl^ represented a logical step. This is supported by previous studies, which demonstrated that *L. lactis* is a good candidate for orthogonal expression of functional modification enzymes, e.g., GdmD of *Staphylococcus gallinarum* (van Heel et al., 2013). This illustrates the high combinatorial possibilities of different PTMs in these organisms and their potential for synthesis of new antibiotics (Montalbán-López et al., 2017). With PylRS–tRNA^Pyl^, we expressed for the first time a tRNA synthetase with its cognate tRNA of archaeal origin in *L. lactis*. The functionality of this orthogonal pair in this host could also be shown in combination with the NisBTC nisin modification enzymes, leading to PTM of the ncAA-modified nisin precursor and transport out of the cell into the culture supernatant. The high substrate tolerance of the NisBTC system allows modification not only of RiPPs from different origins (van Heel et al., 2016), but also of the newly synthesized nisin(BocK) variants. With our results for *L. lactis* and previous works on *B. cereus* (Luo et al., 2016) and *Streptomyces albus* (Lopatniuk et al., 2017), SCS has now been successfully implemented in three Gram-positive organisms from different prokaryotic families. In all three cases, expression of an OTS was successfully combined with the natural PTM enzymes for either nisin (current study), thiocillin (Luo et al., 2016), or cinnamycin (Lopatniuk et al., 2017) of the expression host. These findings indicate the potential of Gram positives as production hosts for new peptide antibiotics. Concerning the location of ncAA incorporation, especially nisinK12 represents an interesting position for mutagenesis. In accordance with our data, previous studies revealed that replacement of K12 by alanine, serine, or threonine improves the antimicrobial activity of nisin against diverse pathogens, e.g., *Enterococcus faecalis*, *B. cereus*, and *S. aureus* (Molloy et al., 2013). The antimicrobial activity depends on the chemistry of the chosen amino acids, since replacement of K12 by aspartate combined with four other replacements of amino acids by negatively charged ones led to a tremendous decrease of the antimicrobial activity (Khusainov and Kuipers, 2013).

The possibility to combine PTM and SCS, either in *E. coli* or in *L. lactis*, is good news for the development of new antimicrobials, since it allows researchers to benefit from several advantages. For both organisms, high- and low-copy vectors with inducible and constitutive promoters are available, allowing fast construction of the aimed construct and a high combinatorial potential (Baumann et al., 2017). A short generation time allows fast experiments and fast results. In special cases, sophisticated expression setups even allow production to outcompete the natural host (Kunji et al., 2003; Sezonov et al., 2007; Ongey and Neubauer, 2016).

In comparative experiments using both bacterial hosts, currently, *E. coli* is still the preferable host, benefitting from years of SCS optimization. However, this can be changed in future given that the OTS efficiency in *L. lactis* can be optimized. As for the sophisticated *E. coli* systems which still hold room for optimization (Zheng et al., 2016), the genetic setup for the archaeal tRNA and its amino acyl tRNA synthetase expression certainly requires fine-tuning and balancing with the three nisin production genes. For example, in *E. coli*, *pylTS* were expressed constitutively and only *nisABC* expression was regulated by the inducible T7 promoter. This potentially led to a higher production yield of nisin(I4BocK) and nisin(K12BocK) in *E. coli* than in *L. lactis*. Additionally, the mass spectrometry data suggest a main population of fully post-translationally modified nisin variants in contrast to the mixed dehydration status resulting from *L. lactis* cultivation. In the latter host, the expression of *pylTS* as well as *nisABTC* was regulated by a P_nisA_ promoter, a method optimized for RiPP synthesis in *L. lactis*. Therefore, different promoter setups and gene copy numbers for *pylTS* as well as RBS and codon optimization of *pylS*, which originates from a genetically distant archaeal host, are conceivable options. In previous works on *B. cereus*, the expression of the orthogonal tRNA was driven by host cell tRNA promoters – a promising setup to be tested using *L. lactis* (Luo et al., 2016).

Additional approaches to further improve the target production and OTS performance can be followed in both hosts: Besides improving the aminoacyl-tRNA synthetase efficiency, rational (re)design of orthogonal tRNAs can allow for more efficient ncAA incorporation (Fan et al., 2015; Maranhao and Ellington, 2017). Genome engineering can allow the knockout of RF-1, which otherwise competes with amber suppressors and leads to translation termination. Accordingly, the resulting *E. coli* K and B strains can enable efficient and multi-site ncAA incorporation (Mukai et al., 2015; Zheng et al., 2016). Moreover, elongation factor Tu (EF-Tu; which delivers the aminoacyl-tRNAs to the ribosome) and other parts the ribosomal machinery can be engineered (Mukai et al., 2017). Little is known about stop codon suppressor mutants in *L. lactis*. Only one instance of an amber suppressor was reported so far (Dickely et al., 1995). This unwanted mutation can in our experiments be excluded by the obtained mass spectrometry data. Additionally, with the first documented OTS shuttle vector system for *E. coli*, *Salmonella enterica*, and *Vibrio cholerae*, new developments also for Gram-positive species are expected in the long run (Volkwein et al., 2017).

Clearly, the prospects are as big as the challenges. Recent reviews highlight the strong pharmaceutical potential of ncAA-supplemented drugs against various pathogens, e.g., MRSA, *Acinetobacter baumannii*, *C. difficile*, and *Pseudomonas aeruginosa* (Blaskovich, 2016; Hicks, 2016; Wang et al., 2016; Baumann et al., 2017). The modification of AMPs with new-to-nature ncAAs presents a versatile tool to fight the increasing lack of antimicrobial drugs (Blaskovich, 2016; Baumann et al., 2017). As the next stage, synthetic cells metabolically engineered to produce ncAAs *in situ* from simple chemical precursors are highly promising, especially for large-scale fermentation in industrial biotechnology (Völler and Budisa, 2017). Genetic code engineering and expansion complement traditional gene modification technologies, breaking the limitations in the number of building blocks and chemical diversity. This synthetic co-translational modification in combination with natural PTM machineries from diverse sources will become the method of choice to synthesize RiPP-based derivatives with novel and emergent properties.

## Author Contributions

OK and NB conceived the study. MB designed and performed the experimental part using *L. lactis* as nisin production host. TB and JN designed and performed the experimental part using *E. coli* as nisin production host. DP and RW designed and produced the amber codon scanned library of *nisA*. MB and TB drafted and wrote the manuscript. OK and NB equally contributed to the revision of the manuscript to obtain the final version. All contributors read and approved the final version of this manuscript.

## Conflict of Interest Statement

The authors declare that the research was conducted in the absence of any commercial or financial relationships that could be construed as a potential conflict of interest.

## Abbreviations

AMP: antimicrobial peptide
BocK: *N*_ε_-Boc-L-lysine
ncAA: non-canonical amino acid
OTS: orthogonal translation system
PTM: post-translational modification
RBS: ribosome-binding site
RiPP: ribosomally synthesized and post-translationally modified peptide
SCS: stop codon suppression
WT: wild-type
