# Supplementary material for: Expanding the Genetic Code of Lactococcus lactis and Escherichia coli to Incorporate Non-canonical Amino Acids for Production of Modified Lantibiotics
Source: Front Microbiol. 2018 Apr 6;9:657. doi: 10.3389/fmicb.2018.00657 (PMC5897534; doi:10.3389/fmicb.2018.00657)
Supplement: Supplementary file 1 [file Presentation_1.PDF]

Supplementary Material

Expanding the genetic code of *Lactococcus lactis* and *Escherichia coli* to incorporate non-canonical amino acids for production of modified lantibiotics

Maike Bartholomae*[1], Tobias Baumann*[2], Jessica H. Nickling[2], David Peterhoff[3], Ralf Wagner[3], Nediljko Budisa#[2], Oscar P. Kuipers#[1]

1: Molecular Genetics Group, Groningen Biomolecular Sciences and Biotechnology Institute, Department of Molecular Genetics, University of Groningen, Nijenborgh 7, 9747 AG Groningen, The Netherlands

2: Biocatalysis Group, Department of Chemistry, Technische Universität Berlin (Berlin Institute of Technology), Müller-Breslau-Str. 10, 10623 Berlin, Germany

3: Institute of Medical Microbiology and Hygiene, University Regensburg, Franz-Josef-Strauß-Allee 11, 93053 Regensburg, Germany

* These authors contributed equally to this work

# Corresponding authors: Oscar P. Kuipers, E-mail: o.p.kuipers@rug.nl or Nediljko Budisa, E-mail: nediljko.budisa@tu-berlin.de

Contents:

1. Supplementary Methods:
    - General cloning procedures
    - Construction of the GFP amber suppression reporter
    - FACS measurements
2. Supplementary Results: Analysis of the GFP amber suppression reporter by FACS
3. Supplementary Table 1: Bacterial strains and plasmids
4. Supplementary Table 2: Oligonucleotides
5. Supplementary Figures
6. Supplementary References

1. Supplementary methods

General cloning procedures

In Supplementary Table 1, all bacterial strains and plasmids of this study are listed. Oligonucleotide primers are given in Supplementary Table 2. To purify plasmid DNA the Nucleospin Extraction Kit (Macherey-Nagel) was used. Restriction enzymes, T4-DNA-ligase and DNA polymerase were utilized according to the manufacturer's instructions. For purification of linear DNA fragments the NucleoSpin® Gel and PCR clean up Kit (Macherey-Nagel) was used. DNA sequencing was carried out by Macrogen (Amsterdam, the Netherlands).

Construction of the GFP amber suppression reporter

To introduce the amber codon at position N149 in *gfp*, a two-step mutagenesis PCR setup was used with a forward primer containing TAG at this position. The reverse primer GFPrevXba introduced the XbaI restriction site. The resulting PCR product was utilized as primer for the second PCR with GFPfwKpn as forward primer to introduce the KpnI restriction site. The final product was digested with KpnI and XbaI as well as pNZ-RBSpylTS. The resulting vectors was named pNZ-RBSpylTSGFP(N149amber).

FACS measurements

To determine the intact cell fluorescence, *L. lactis* overnight cultures were transferred to fresh CDM and grown at 30 ˚C to an OD_{600} of 0.4. Three setups were analyzed: An uninduced control sample, one sample induced with 10 ng/ml nisin and one sample with 10 ng/ml nisin and 1 mM BocK. At distinct time points after induction, 200 µl (approximately 50000) cells were mixed with PBS and the GFP fluorescence was analyzed on a BD FACSCanto instrument (BD Biosciences) using a 488 nm argon laser. Wild-type GFP samples served as positive control. FACSDiva Software (BD Biosciences) was used for data collection. Subsequently, the data were analyzed with FCSalyzer 0.9.13-alpha (https://sourceforge.net/projects/fcsalyzer/).

2. Supplementary results

Analysis of the GFP amber suppression reporter by FACS

To test the functionality of PylRS-tRNA^{Pyl} in *L. lactis* by determining GFP fluorescence, the codon at location N149 of *gfp* was replaced by TAG. The chosen amino acid replacement is equivalent to the location previously tested using *E. coli* as host (Exner et al., 2017; Mu et al., 2013). The functionality of the chosen GFP variant was tested successfully in *L. lactis*. The expression of PylRS-tRNA^{Pyl}, wild-type GFP and GFP(amber) was regulated by P_{nisA} and induced by addition of nisin. The fluorescence resulting from expression of *gfp*(N149amber) in the presence and absence of BocK was determined by FACS. Cells expressing wild-type GFP served as positive control, cells expressing a construct lacking GFP and expressing only the PylRS-tRNA^{Pyl}-pair were utilized as negative control. Repeated measurements over several hours after induction of expression revealed a small, but reproducible increase in fluorescence for the GFP(N149BocK) variant in contrast to the negative control as shown in Supplementary Fig. 1 with an exemplary measurement after 2 h.

Supplementary Table 1: Bacterial strains and plasmids

**Strain/plasmid**	**Genotype/function**	**Reference**
L. lactis NZ9000	MG1363 Δ*pepN*::*nisRK*	(Kuipers et al., 1998)
L. lactis NZ9000 pNZnisPT pIL253	Cm^R, Ery^R, nisin-sensitive indicator strain expressing NisPT	(Khusainov et al., 2011)
pET-21a_P_His6_leader_nisA	Amp^R, pBR322 origin, *E. coli* expression vector for *nisA*(amber) stop codon library members with nisin leader carrying an N-terminal His-tag	This study
pIL3EryBTC	Ery^R, *nisBTC* expression vector for modification and transport of lantibiotics in *L. lactis*	(Rink et al., 2005)
pJZ_Ptrp_pylT_strep-MmPylS(Y384F)	Cm^R, p15A origin, template for amplifying *Methanosarcina mazei pylS*. OTS (*pylTS*) expression is driven from an *E. coli* trp promoter (Ptrp)	(Exner et al., 2017)
pJZ_Ptrp_pylT_MmPylS (Y384F) PT7 nisA(amber)	pJZ_Ptrp_pylT_strep-MmPylS(Y384F) derivative with T7 promoter driving *nisA*(amber) expression in *E. coli*	This study
pRSFDuet-1 nisBC	nisin PTM machinery for NisBC expression in *E. coli*	(Baumann et al., 2017)
pNZ8048	Cm^R, nisin-inducible P_{nisA} for gene expression in *L. lactis*	(de Ruyter et al., 1996)
$pLG_{\Delta RBS}$-GFP	pNZ8048 derivative, RBS upstream GFP mutated	(van Gijtenbeek et al., 2016)
pNZ8048G	pNZ8048 derivative, expression of GFP in *L. lactis*	(Mu et al., 2013)
pNZ-RBSpylTS	Derivative of $pLG_{\Delta RBS}$-GFP, expression of PylRS-$tRNA^{Pyl}$ orthogonal translation system in *L. lactis*	This study
pNZ-RBSpylTSnisA(I4amber)	Derivative of pNZ-RBSpylTS, expression of *nisA*, triplet encoding I4 replaced by TAG	This study
pNZ-RBSpylTSnisA(K12amber)	Derivative of pNZ-RBSpylTS, expression of *nisA*, triplet encoding K12 replaced by TAG	This study
pNZ-RBSpylTSnisA	Derivative of pNZ-RBSpylTS, expression of *nisA* wild-type	This study
pNZ-RBSpylTSGFP(N149amber)	Derivative of pNZ-RBSpylTS, expression of GFP, triplet encoding N149 replaced by TAG	This study

Supplementary Table 2: Oligonucleotides

Oligonucleotide	Sequence (5'→3')	Purpose
GFPfwNco	ATAAGAACACCATGGACCATGGGAAAAGGAGAAG	forward primer to amplify GFP including NcoI restriction site
GFP149TAGmutfw	CAGCCACTAGGTATACATCACTGCTGAC	forward primer to mutate *gfp* N149 codon to TAG
GFPrevHind	ATGATCAAGAAGCTTTTACTTATAAAGCTCATCCAT GC	reverse primer to amplify *gfp* including a HindIII restriction site
NisAWTrevXba	AGAGAACAGAGTCTAGATGCTCGAGTTATTTGCTTA CGTGAATAC	Construction of pNZ8048pylSnisA
NisLibraryfwKpn	AGAAGAGGTACCGAAGGAGATATACATATGGGCAG CAG	Construction of pNZ8048pylSnisA(amber)
NisLibraryrevXba	ACGATATCTAGACCGGATCTCAGTGGTGGTG	Construction of pNZ8048pylSnisA(amber)
PylTSfwNco	CATGATATACCATGGGGAAACCTGATCATGTAGATC	Construction of pNZ8048pylTS
PylTSrevKpn	ATCGTCATCGGTACCTTACAGGTTGGTAGAAATCC	Construction of pNZ8048pylTS
PT7-NisA_f	CTAACTAGTGCGTAGAGGATCGAGATCTC	Construction of pJZ_Ptrp_pylT_MmPylS(Y384F) PT7 nisA(amber)
NisA T7term_r	GCTCTGCAGAGGTCCCATTCGCCAATCCGG	Construction of pJZ_Ptrp_pylT_MmPylS(Y384F) PT7 nisA(amber)
T7-fw	TAATACGACTCACTATAGGG	*nisA*(amber) library construction
T7-rev	GCTAGTTATTGCTCAGCGG	*nisA*(amber) library construction
Nis_TAG_01_fwd	GGTGCATCACCACGCTAGACAAGTATTTCGCTA	*nisA*(amber) library construction
Nis_TAG_01_rev	TAGCGAAATACTTGTCTAGCGTGGTGATGCACC	*nisA*(amber) library construction
Nis_TAG_02_fwd	GCATCACCACGCATTTAGAGTATTTCGCTATGT	*nisA*(amber) library construction
Nis_TAG_02_rev	ACATAGCGAAATACTCTAAATGCGTGGTGATGC	*nisA*(amber) library construction
Nis_TAG_03_fwd	TCACCACGCATTACATAGATTTCGCTATGTACA	*nisA*(amber) library construction
Nis_TAG_03_rev	TGTACATAGCGAAATCTATGTAATGCGTGGTGA	*nisA*(amber) library construction
Nis_TAG_04_fwd	CCACGCATTACAAGTTAGTCGCTATGTACACCC	*nisA*(amber) library construction
Nis_TAG_04_rev	GGGTGTACATAGCGACTAACTTGTAATGCGTGG	*nisA*(amber) library construction
Nis_TAG_05_fwd	CGCATTACAAGTATTTAGCTATGTACACCCGGT	*nisA*(amber) library construction
Nis_TAG_05_rev	ACCGGGTGTACATAGCTAAATACTTGTAATGCG	*nisA*(amber) library construction
Nis_TAG_06_fwd	ATTACAAGTATTTCGTAGTGTACACCCGGTTGT	*nisA*(amber) library construction
Nis_TAG_06_rev	ACAACCGGGTGTACACTACGAAATACTTGTAAT	*nisA*(amber) library construction
Nis_TAG_07_fwd	ACAAGTATTTCGCTATAGACACCCGGTTGTAAA	*nisA*(amber) library construction
Nis_TAG_07_rev	TTTACAACCGGGTGTCTATAGCGAAATACTTGT	*nisA*(amber) library construction
Nis_TAG_08_fwd	AGTATTTCGCTATGTTAGCCCGGTTGTAAAACA	*nisA*(amber) library construction
Nis_TAG_08_rev	TGTTTTACAACCGGGCTAACATAGCGAAATACT	*nisA*(amber) library construction
Nis_TAG_09_fwd	ATTTCGCTATGTACATAGGGTTGTAAAACAGGA	*nisA*(amber) library construction
Nis_TAG_09_rev	TCCTGTTTTACAACCCTATGTACATAGCGAAAT	*nisA*(amber) library construction
Nis_TAG_10_fwd	TCGCTATGTACACCCTAGTGTAAAACAGGAGCT	*nisA*(amber) library construction
Nis_TAG_10_rev	AGCTCCTGTTTTACACTAGGGTGTACATAGCGA	*nisA*(amber) library construction
Nis_TAG_11_fwd	CTATGTACACCCGGTTAGAAAACAGGAGCTCTG	*nisA*(amber) library construction
Nis_TAG_11_rev	CAGAGCTCCTGTTTTCTAACCGGGTGTACATAG	*nisA*(amber) library construction
Nis_TAG_12_fwd	TGTACACCCGGTTGTTAGACAGGAGCTCTGATG	*nisA*(amber) library construction
Nis_TAG_12_rev	CATCAGAGCTCCTGTCTAACAACCGGGTGTACA	*nisA*(amber) library construction

Nis_TAG_13_fwd	ACACCCGGTTGTAAATAGGGAGCTCTGATGGGT	*nisA*(amber) library construction
Nis_TAG_13_rev	ACCCATCAGAGCTCCCTATTTACAACCGGGTGT	*nisA*(amber) library construction
Nis_TAG_14_fwd	CCCGGTTGTAAAACATAGGCTCTGATGGGTTGT	*nisA*(amber) library construction
Nis_TAG_14_rev	ACAACCCATCAGAGCCTATGTTTTACAACCGGG	*nisA*(amber) library construction
Nis_TAG_15_fwd	GGTTGTAAAACAGGATAGCTGATGGGTTGTAAC	*nisA*(amber) library construction
Nis_TAG_15_rev	GTTACAACCCATCAGCTATCCTGTTTTACAACC	*nisA*(amber) library construction
Nis_TAG_16_fwd	TGTAAAACAGGAGCTTAGATGGGTTGTAACATG	*nisA*(amber) library construction
Nis_TAG_16_rev	CATGTTACAACCCATCTAAGCTCCTGTTTTACA	*nisA*(amber) library construction
Nis_TAG_17_fwd	AAAACAGGAGCTCTGTAGGGTTGTAACATGAAA	*nisA*(amber) library construction
Nis_TAG_17_rev	TTTCATGTTACAACCCTACAGAGCTCCTGTTTT	*nisA*(amber) library construction
Nis_TAG_18_fwd	ACAGGAGCTCTGATGTAGTGTAACATGAAAACA	*nisA*(amber) library construction
Nis_TAG_18_rev	TGTTTTCATGTTACACTACATCAGAGCTCCTGT	*nisA*(amber) library construction
Nis_TAG_19_fwd	GGAGCTCTGATGGGTTAGAACATGAAAACAGCA	*nisA*(amber) library construction
Nis_TAG_19_rev	TGCTGTTTTCATGTTCTAACCCATCAGAGCTCC	*nisA*(amber) library construction
Nis_TAG_20_fwd	GCTCTGATGGGTTGTTAGATGAAAACAGCAACT	*nisA*(amber) library construction
Nis_TAG_20_rev	AGTTGCTGTTTTCATCTAACAACCCATCAGAGC	*nisA*(amber) library construction
Nis_TAG_21_fwd	CTGATGGGTTGTAACTAGAAAACAGCAACTTGT	*nisA*(amber) library construction
Nis_TAG_21_rev	ACAAGTTGCTGTTTTCTAGTTACAACCCATCAG	*nisA*(amber) library construction
Nis_TAG_22_fwd	ATGGGTTGTAACATGTAGACAGCAACTTGTCAT	*nisA*(amber) library construction
Nis_TAG_22_rev	ATGACAAGTTGCTGTCTACATGTTACAACCCAT	*nisA*(amber) library construction
Nis_TAG_23_fwd	GGTTGTAACATGAAATAGGCAACTTGTCATTGT	*nisA*(amber) library construction
Nis_TAG_23_rev	ACAATGACAAGTTGCCTATTTCATGTTACAACC	*nisA*(amber) library construction
Nis_TAG_24_fwd	TGTAACATGAAAACATAGACTTGTCATTGTAGT	*nisA*(amber) library construction
Nis_TAG_24_rev	ACTACAATGACAAGTCTATGTTTTCATGTTACA	*nisA*(amber) library construction
Nis_TAG_25_fwd	AACATGAAAACAGCATAGTGTCATTGTAGTATT	*nisA*(amber) library construction
Nis_TAG_25_rev	AATACTACAATGACACTATGCTGTTTTCATGTT	*nisA*(amber) library construction
Nis_TAG_26_fwd	ATGAAAACAGCAACTTAGCATTGTAGTATTCAC	*nisA*(amber) library construction
Nis_TAG_26_rev	GTGAATACTACAATGCTAAGTTGCTGTTTTCAT	*nisA*(amber) library construction
Nis_TAG_27_fwd	AAAACAGCAACTTGTTAGTGTAGTATTCACGTA	*nisA*(amber) library construction
Nis_TAG_27_rev	TACGTGAATACTACACTAACAAGTTGCTGTTTT	*nisA*(amber) library construction
Nis_TAG_28_fwd	ACAGCAACTTGTCATTAGAGTATTCACGTAAGC	*nisA*(amber) library construction
Nis_TAG_28_rev	GCTTACGTGAATACTCTAATGACAAGTTGCTGT	*nisA*(amber) library construction
Nis_TAG_29_fwd	GCAACTTGTCATTGTTAGATTCACGTAAGCAAA	*nisA*(amber) library construction
Nis_TAG_29_rev	TTTGCTTACGTGAATCTAACAATGACAAGTTGC	*nisA*(amber) library construction
Nis_TAG_30_fwd	ACTTGTCATTGTAGTTAGCACGTAAGCAAATAA	*nisA*(amber) library construction
Nis_TAG_30_rev	TTATTTGCTTACGTGCTAACTACAATGACAAGT	*nisA*(amber) library construction
Nis_TAG_31_fwd	TGTCATTGTAGTATTTAGGTAAGCAAATAACTC	*nisA*(amber) library construction
Nis_TAG_31_rev	GAGTTATTTGCTTACCTAAATACTACAATGACA	*nisA*(amber) library construction
Nis_TAG_32_fwd	CATTGTAGTATTCACTAGAGCAAATAACTCGAG	*nisA*(amber) library construction
Nis_TAG_32_rev	CTCGAGTTATTTGCTCTAGTGAATACTACAATG	*nisA*(amber) library construction
Nis_TAG_33_fwd	TGTAGTATTCACGTATAGAAATAACTCGAGCAC	*nisA*(amber) library construction
Nis_TAG_33_rev	GTGCTCGAGTTATTTCTATACGTGAATACTACA	*nisA*(amber) library construction
Nis_TAG_34_fwd	AGTATTCACGTAAGCTAGTAACTCGAGCACCAC	*nisA*(amber) library construction

Nis_TAG_34_rev	GTGGTGCTCGAGTTACTAGCTTACGTGAATACT	*nisA*(amber) library construction

Supplementary Figures

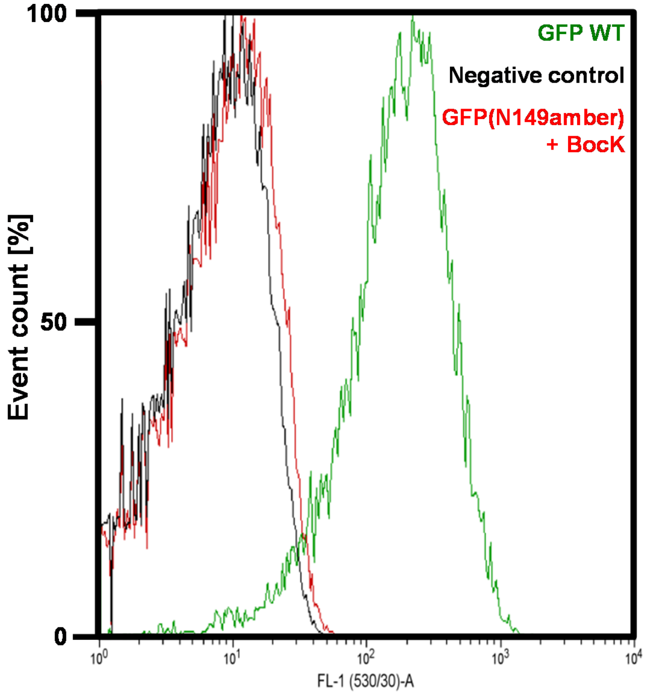

Supplementary Figure 1. FACS analysis to determine incorporation of BocK into the GFP amber suppression reporter construct. To determine the functionality of the PylRS-based OTS in *L. lactis,* an expression cassette for *gfp*(N149amber) under control of P_{nisA} was constructed and used to create plasmid vector pNZ-RBSpylTSGFP(N149amber). Cells were grown with and without BocK supplementation to determine the intact cell fluorescence by FACS. Cells expressing wild-type GFP served as positive control (green), cells bearing only the PylRS-tRNA^{Pyl} construct as negative control (black curve). The expression of PylRS-tRNA^{Pyl} and GFP(N149amber) was induced with nisin and fluorescence of the cells analyzed for several hours after induction. Data from 2 h after induction is shown in the histogram. A small, but reproducible increase in fluorescence was detectable when the ncAA was supplied (red).

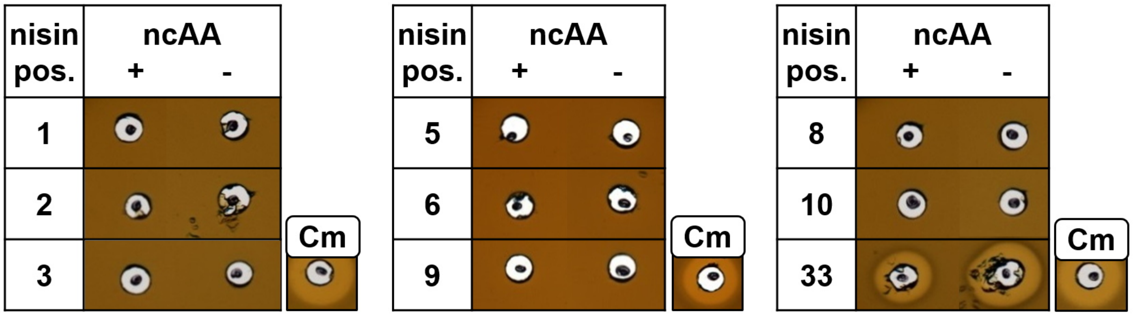

Supplementary Figure 2. Antimicrobial activity of recombinant nisin expression samples produced by SCS in *E. coli*. Activity was determined against the nisin-sensitive strain *L. lactis* NZ9000 pNZnisPT pIL253 (this strain expresses NisP, which catalyzes removal of the nisin leader peptide). The *nisA*(amber) variants were expressed in presence or in absence of the ncAA BocK. 50 µL *E. coli* cell lysate normalized relative to the harvested cell density were used. Cm served as antimicrobial control compound. For each of the three groups, all image sections including the Cm control originate from the same assay plate.

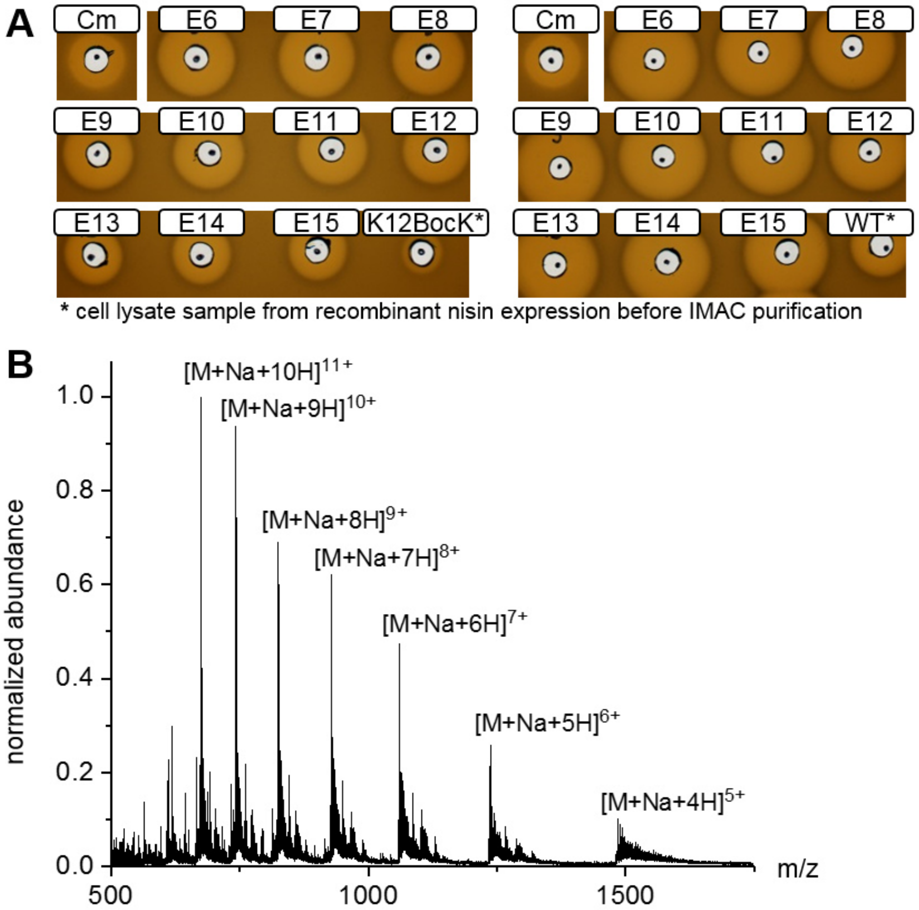

Supplementary Figure 3. Activity and LC-MS analysis of IMAC-purified nisin samples. **(A)** Antimicrobial activity of nisin samples produced in *E. coli* before and after IMAC purification. The left panel shows nisin(K12BocK) samples where expression was conducted in presence of the ncAA BocK. The right panel shows recombinant wild-type nisin samples. *E. coli* cell lysates were prepared as above. For both genetic setups, corresponding cell lysate (*) and IMAC elution fraction samples (labeled "E") were used. Activity was determined against the nisin-sensitive strain *L. lactis* NZ9000 pNZnisPT pIL253 (this strain expresses NisP, which catalyzes removal of the nisin leader peptide). Cm served as antimicrobial control compound. Relative to the cell lysate samples before purification, inhibition halos of IMAC-purified samples are larger. Pure IMAC elution buffer did not result in antimicrobial activity (data not shown). For each of the two panels, all image sections originate from the same assay plate. **(B)** LC-MS deconvolution chromatogram for nisin(K12BocK) purified via IMAC. See Fig. 1B inset for detailed deconvolution data and theoretical masses.

Supplementary References

Baumann, T., Nickling, J. H., Bartholomae, M., Buivydas, A., Kuipers, O. P., and Budisa, N. (2017). Prospects of *In vivo* Incorporation of Non-canonical Amino Acids for the Chemical Diversification of Antimicrobial Peptides. *Front. Microbiol.* 8, 124. doi:10.3389/fmicb.2017.00124.

Exner, M. P., Kuenzl, T., To, T. M. T., Ouyang, Z., Schwagerus, S., Hoesl, M. G., et al. (2017). Design of S-Allylcysteine in Situ Production and Incorporation Based on a Novel Pyrrolysyl-tRNA Synthetase Variant. *Chembiochem* 18, 85–90. doi:10.1002/cbic.201600537.

van Gijtenbeek, L. A., Robinson, A., van Oijen, A. M., Poolman, B., and Kok, J. (2016). On the Spatial Organization of mRNA, Plasmids, and Ribosomes in a Bacterial Host Overexpressing Membrane Proteins. *PLoS Genet.* 12, e1006523. doi:10.1371/journal.pgen.1006523.

Khusainov, R., Heils, R., Lubelski, J., Moll, G. N., and Kuipers, O. P. (2011). Determining sites of interaction between prenisin and its modification enzymes NisB and NisC. *Mol. Microbiol.* 82, 706–718. doi:10.1111/j.1365-2958.2011.07846.x.

Kuipers, O. P., de Ruyter, P. G. G. ., Kleerebezem, M., and de Vos, W. M. (1998). Quorum sensing-controlled gene expression in lactic acid bacteria. *J. Biotechnol.* 64, 15–21. doi:10.1016/S0168-1656(98)00100-X.

Mu, D., Montalbán-López, M., Masuda, Y., and Kuipers, O. P. (2013). Zirex: a novel zinc-regulated expression system for *Lactococcus lactis*. *Appl. Environ. Microbiol.* 79, 4503–8. doi:10.1128/AEM.00866-13.

Rink, R., Kuipers, A., de Boef, E., Leenhouts, K. J., Driessen, A. J. M., Moll, G. N., et al. (2005). Lantibiotic structures as guidelines for the design of peptides that can be modified by lantibiotic enzymes. *Biochemistry* 44, 8873–82. doi:10.1021/bi050081h.

de Ruyter, P. G., Kuipers, O. P., and de Vos, W. M. (1996). Controlled gene expression systems for *Lactococcus lactis* with the food-grade inducer nisin. *Appl. Environ. Microbiol.* 62, 3662–7. Available at: http://aem.asm.org/content/62/10/3662.abstract.
